# RISK PERCEPTIONS OF DIRECT CARE NURSING HOME STAFF ASSOCIATED WITH RESTRICTED SOCIAL ACTIVITIES

**DOI:** 10.1093/geroni/igae098.0080

**Published:** 2024-12-31

**Authors:** Nahida Akter, Melissa McClean, Kalei Kowalchik, Jacqueline Mogle, Joan Carpenter, LIza Behrens

**Affiliations:** The Pennsylvania State University, University Park, Pennsylvania, United States; University of Maryland Baltimore, Baltimore, Maryland, United States; The Pennsylvania State University, University Park, Pennsylvania, United States; Clemson University, Clemson, South Carolina, United States; University of Maryland Baltimore, Baltimore, Maryland, United States; The Pennsylvania State University, University Park, Pennsylvania, United States

## Abstract

Recognized globally, social activities support better quality of life in older adulthood. The COVID-19 pandemic presented unprecedented challenges for nursing homes (NHs) in balancing infection prevention control (IPC) measures with honoring resident preferences for social activities. The purpose of this study was to explore NH staff’s risk perceptions around facilitating resident preferences amidst pandemic restrictions. Guided by the Preference-Based Person-Centered Risk Engagement Model, this study used mixed methods to achieve an in-depth understanding NH staff risk perception. Quantitative data included demographics and risk perceptions using the Risk Propensity Scale. Participants (N=24) were 39 years on average (range: 18-62), mostly female (88%) and white (79%). Purposive sampling reflected a range of roles within the NH context: certified nursing assistants (29%), activities directors (25%), social workers (25%), and nurses (21%). Average risk perceptions scores were in the risk avoider range at 3.53 (SD = 1.43; range: 1.00-7.14) and most participants (58%) self-identified as risk avoiders. Those who identified as risk seekers were significantly more likely to be male (chi-sq(2)=17.14, p<.01) and African American (chi-sq(4)=20.42, p<.01). Qualitatively, semi-structured individual interviews collected staff perceptions of risk related to residents’ social activities. Guided content analysis revealed two main themes supported by six sub-themes: Factors of decision-making (family influence, organizational, staff and resident characteristics) and Direct care staff influencing preference-based care (cognitive skills and technical skills). Findings will be discussed considering implications for how best to support direct-care staff with varied perceptions of risk (risk seekers/risk avoiders) to encourage social activities when IPC measures are indicated.

